# Analysis of the Six Sigma Principle in Pre-analytical Quality for Hematological Specimens

**DOI:** 10.7759/cureus.42434

**Published:** 2023-07-25

**Authors:** Queen Mary A, Subhashish Das, Nikhil Chaudhary, Kalyani Raju

**Affiliations:** 1 Pathology, Sri Devaraj Urs Medical College, Kolar, IND

**Keywords:** pareto chart, laboratory, blood, six sigma, quality

## Abstract

Introduction

Blood tests are essential for detecting and treating hospitalized individuals with diseases. Laboratory blood tests provide doctors with critical information required to treat their patient's illnesses. The most common sources of error in clinical laboratories are pre-analytical errors. Although quality control measures can remediate analytical errors, there is a requirement for stringent quality checks in the pre-analytical sector as these activities are performed outside of the laboratory. Pre-analytical errors when combined with the sigma value can reflect a better picture as the sigma value represents the laboratory's performance.

Aim

In this study, six sigma and the Pareto principle were utilized to assess pre-analytical quality indicators for evaluating the performance of a clinical hematology laboratory.

Methodology

This is a retrospective observational study conducted from 2015 to 2023 (for a period of eight years). Information about the frequency of pre-analytical errors was retrieved from the hematology section of the central diagnostic research laboratory information system and the data was entered into an MS Excel sheet and data was evaluated utilizing SPSS version 23 (IBM Corp., Armonk, NY).

Results

In the current research, total of 15 pre-analytical errors were noted. Out of the total 15 pre-analytical errors studied, 55.4% of pre-analytical errors were noted among which 80% errors were due to lack of mention of sample type or received time and 20% of errors were attributed to no mention of diagnosis in requisition forms. The next most common errors noted were insufficient samples (8.26%) followed by absence of physician’s signature (7%), incomplete request form (5.4%), age (4.2%), unique hospital identification (UHID) number (3.7%), clotted samples and transportation of the samples (3.6%), date and incorrect vials (2.6%). Gender (0.95%), hemolysed (0.85%), and lipemic samples (0.45%). Hemolysed and lipemic samples had a sigma value of 4.4 and 4.6, respectively, whereas gender and age had a sigma value of 4.3 and 3.8, inadequate sample for testing and an incorrect anticoagulant to blood ratio had a sigma value of 3.6, indicating that sample collection has to be improved as the inverse relationship is noted between sigma value and laboratory performance.

Conclusion

Pareto chart and sigma value can help recognize most common pre-analytical errors, which consequently will help to prevent further recurrence of pre-analytical errors. Adequate training with regard to best practices in phlebotomy for interns, clinicians and technicians must be provided to decrease quantitative errors, which will further enhance total quality management in the laboratory.

## Introduction

One of the most common procedures performed in any modern hospital is blood sampling. If the blood sampling method fails, the healthcare organization may have to repeat the process leading to additional expenditure. So, these failures are known as pre-analytical errors (PAEs) which frequently occur in the pre-testing phase before the blood sample enters the laboratory [1]. The blood test procedure is divided into three stages: the pre-testing phase, the testing phase, and the post-testing phase. The majority of blood test errors arise during the pre-testing phase. Such (PAE) can jeopardize a patient's safety by delaying the clinical decision for further treatment and also it causes discomfort to patients due to repeated withdrawal of blood samples [2]. When compared to other medical fields, error rates in laboratory medicine are modest [3]. Maximum errors occur during the pre-testing phase, it is estimated to be roughly around 70% of all laboratory errors. The pre-testing phase is initiated with the raising of test prescription followed by sample collection, storage, and transportation to the respective laboratory section ultimately leading to the culmination of the pre-testing phase [4]. Failure of PAE has adverse effects on good laboratory practices with poor clinical outcomes. PAE causes blood samples to be rejected by laboratories due to errors such as hemolysis, clotting, unfilled or incorrect tubes, missing samples etc. Several clinical laboratory investigations from recent decades had reported regarding PAE frequency which range from 8% to 14% indicating an increasing interest in this research topic [5,6]. So, it is important to assess the “financial implications” of various laboratory processes to ensure optimal utilization of the available economic resources because for healthcare organizations laboratory costs account for around 5% of the overall budget [7,8]. The usage of the quality instrument six sigma metric has proven to be an encouraging method for refining of pre-analytical phase. This tool is a fundamental measurement that identifies flaws in the process. The six-sigma metric system has a scale of zero to six [9]. A process's minimum acceptable quality level is three, which translates to 66,807 faults per million. The highest quality level, a performance of six, has only 3.4 faults per million or a success percentage of 99.99%. Other technological advancements and quality control methods have resulted in decreasing laboratory mistake rates in recent decades [10]. As a result, in the current study, pre-analytical mistakes were noted at the time of sample collection and in test requisition forms (TRFs), a sigma value was generated for each PAE [11].

## Materials and methods

This was a retrospective observational study done from February 2015 to April 2023 for a duration of eight years. Ethical clearance was obtained from the Institutional ethical clearance committee with IEC no DMC/KLR/IEC/138/2023-24. The hospital provides a wide range of specialty and super-specialty departments. In the Hematology laboratory, approximately 6,000 test request forms and 9,000 to 11,000 blood samples were received from outpatient (OP) and inpatient departments (IPD). Information about the frequency of PAE was retrieved from the hematology Section of the Central diagnostic research laboratory. The data was used retrospectively in a register-based study to answer the research question. The data contained information about blood analysis sent to the hospital laboratory’s section of hematology. The present study was carried out in a local population of approximately about 2,000,000. Samples are collected in appropriately colored vacutainers with proper maintenance of the “cold chain” in order to maintain minimize errors and fulfill quality requirements to maintain proper accountability and traceability. After obtaining the sample in the laboratory, technicians and the section in charge noted any pre-analytical flaws in the request forms and samples. When an error was discovered, it was noted down in the register and an Excel sheet was designated for PAEs. PAEs in the current research were of two categories, namely, sample collection and pre-requisition forms. PAE in sample collection includes an error in the transportation of samples, incorrect vials, insufficient samples, clotted samples, and hemolysed and lipemic samples, whereas PAEs in test requisition forms include an error in unique hospital identification (UHID), errors in date, age, gender, clinical diagnosis, absence of physician signature, incomplete mentioning of the test, incomplete details about inpatient or OP. Data were collected and entered in an Excel sheet. Data were evaluated utilizing SPSS version 23 (IBM Corp., Armonk, NY). Mean, standard deviation, percentage, and frequency were applied for descriptive statistical values. An independent t-test was utilized for inferential statistics. The level of significance was considered 0.05 at a 95% confidence interval. MS Excel and MS Word were utilized to attain numerous types of graphs.

## Results

**Figure 1:**
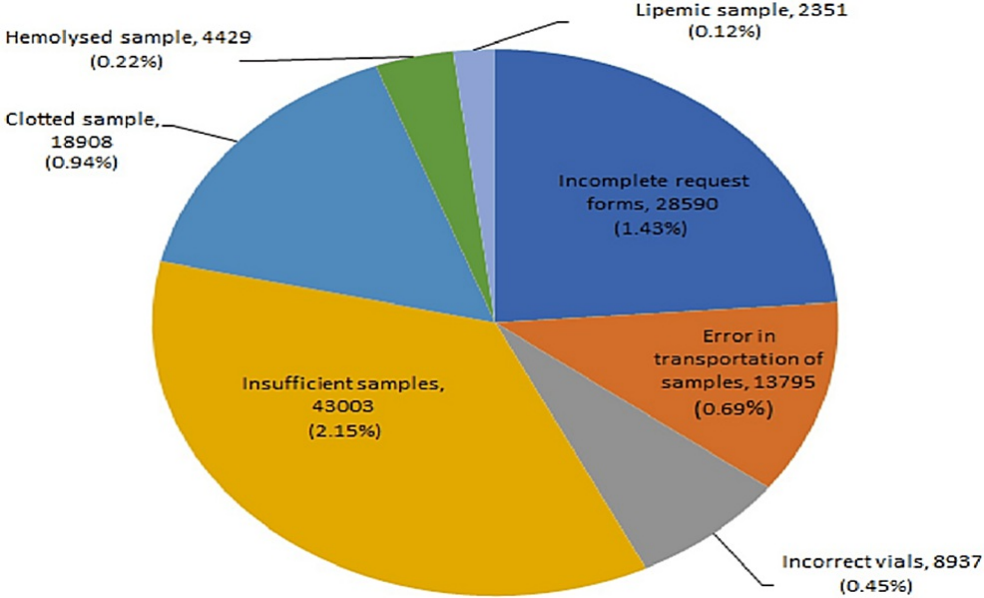
Distribution of pre-analytical error in sample collection.

**Figure 2:**
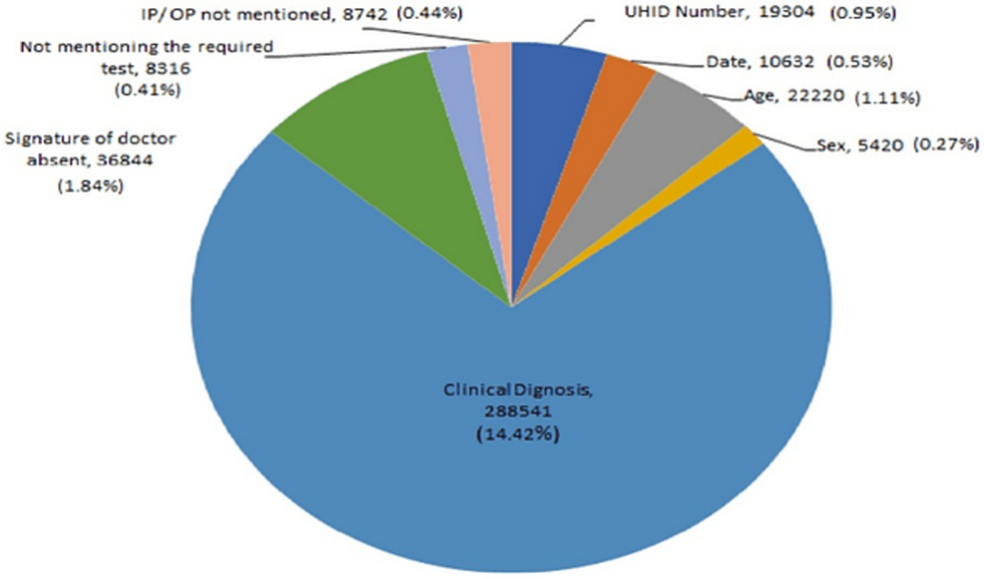
Distribution of pre-analytical errors with respect to test requisition form. Total 19.53% errors (approximately 20% errors). N - Number of pre-analytical errors, UHID - Unique hospital identification number, IP/OP - Inpatient/Outpatient

In the current research, a total of 15 PAEs were noted. Out of total 15 PAEs studied, out of which seven PAEs were noted in sample collection whereas eight PAEs were noted in test requisition form. The commonest PAE with respect to sample collection was noted in insufficient samples (2.14%) and the least percentage of error was noted due to lipemic samples (0.12%) (Figure 1). The commonest PAE with regards to test requisition form was noted in no mention of clinical diagnosis (14.42%) and the least percentage of error was noted due to non-mentioning of gender (0.27%) (Figure 2).

Out of 200,000 samples evaluated following results were noted: 28,590 (1.42%) incomplete request forms were found to have errors, whereas 13,795 (0.69%) errors were found in transportation of samples, 8,937 (0.45%) errors due to incorrect vials, 43,003 (2.14%) errors were due to insufficient samples, 18,908 (0.94%) errors were due to clotted samples, 4,428 (0.22%) errors were due to hemolysed samples and 2,351 (0.12%) errors were found in lipemic samples.

Out of 200,000 samples evaluated following results were noted: 19,304 (0.96%) incomplete request forms were found to have errors in UHID number, whereas 10,632 (0.53%) errors were found in date of the samples, 22,220 (1.11%) errors were observed in the age of the patients, 5,420 (0.27%) errors were observed in gender of the patients, 288,541 (14.42%) errors were observed in clinical diagnosis, 36,844 (1.84%) forms were noted in forms which were without the physician’s signature, 8,316 (0.41%) were found to be incomplete without the name of the test and 8,742 (0.43%) forms were without the inpatient or outpatient details of the patients.

**Figure 3:**
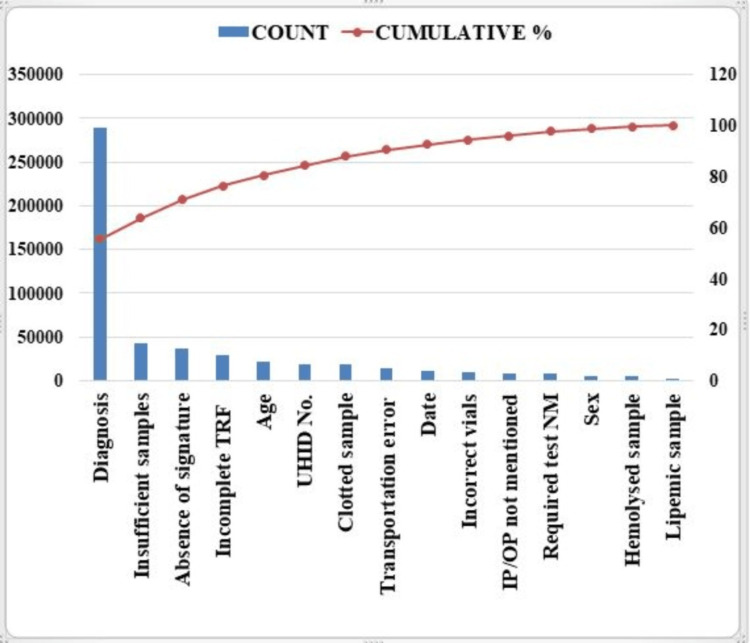
Pareto chart shows both bars and lines represented in same graph and individual errors are represented in descending order by bars and cumulative total by the line. TRF - Test requisition form, UHID - Unique hospital identification number, IP/OP - inpatient/outpatient, NM - Not mentioned

Figure 3 depicts that following calculation of percentage of PAEs, total percentage was noted and plotting of Pareto‘s chart was done for all the 15 PAEs. In the following pareto chart, individual errors are represented in descending order by blue colored bars and cumulative total by the red line which suggests commonest error was too due to improper/non-mention of diagnosis and least common PAE was due to lipemic samples.

Table 1 shows defects per million (DPM) and six sigma values for all the (total 15) PAEs inclusive of errors in sample collections and test requisition form. A sigma value of 3.7 for incomplete request forms was noted, sigma value of 4 was noted for error in transportation of the samples, sigma value of 4.2 was noted for incorrect vials, sigma value of 3.6 was noted for insufficient samples, sigma value of 3.9 was observed for clotted samples, sigma value of 4.4 was noted for hemolysed samples, sigma value of 4.6 was observed for lipemic samples, sigma value of 3.9 was noted for UHID number, sigma value of 4.1 was observed for date, sigma value of 3.8 was noted for age, sigma value of 4.3 was noted for the gender, sigma value of 2.6 was noted for clinical diagnosis, sigma value of 3.6 was observed for the absence of physician’s signature, and sigma value of 4.2 was noted for the absence of the test name and sigma value of 4.2 was noted for incomplete details regarding whether the patient is an inpatient or outpatient. The number of defects seen, and the sample size were noted in Westgard online formula which calculated DPM and six sigma for each PAE was calculated (www.westgard.com/six-sigma-calculators). So, these results suggests that special attention needs to be given to those pre-analytical parameters where decreased sigma value was noted to further enhance the performance and quality in the laboratory as there is inverse relationship between sigma value with regards to quality and performance of laboratory. 

**Table 1:** Six sigma values for pre-analytical quality indicators DPM - Defects per million, OP/IP - Outpatient/Inpatient, UHID - Unique hospital identification number

	DPM	Sigma Value
Incomplete request forms	14,294	3.7
Error in transportation of samples	68,967	4
Incorrect vials	4,468	4.2
Insufficient samples	21,500	3.6
Clotted sample	9,453	3.9
Hemolysed sample	2,214	4.4
Lipemic sample	1,175	4.6
UHID NO	9,651	3.9
Date	5,316	4.1
Age	11,109	3.8
Sex	27,010	4.3
Clinical diagnosis	144,259	2.6
Signature of doctor absent	18,421	3.6
Not mentioning the required test	4,158	4.2
IP/OP not mentioned	4,370	4.2

## Discussion

Laboratory data are crucial for diagnosis and management of disease. The laboratories main focus is to ensure accuracy especially in analytical portion of the testing process but pre-analytical and post-analytical phases both are equally important. Pre-analytical phase is loaded with difficulties in the form of incorrectly filled out requisition forms, lack of trained staff with respect to appropriate phlebotomy practices and so on. Several studies have indicated that the majority of inaccurate results occur during the pre-analytical phase [7,8]. In the present research, the pre-analytical lapses were around 70% which have major clinical and economic consequences, and it affects further management. As a result, the pre-analytical process must be strictly monitored at all times to ensure that laboratory quality meets a standard. Quality indicators (QIs) act as critical performance indicators for the evaluation of the testing process [12]. In the current study, the final consequences of PAE included considerable annual costs [approximately 9,140,000 rupees] that were primarily related to personnel and hospitalization costs. Focus group interviews revealed that the blood sampling procedure was challenging further creating feelings of stress and frustration [13]. Six sigma is regarded as a vital instrument in the process of sustaining refined and sustained laboratory quality standards. The lean six sigma approach strives to reduce unproductive actions during sample processing. When the sigma metric is 6, the process is stated to have only 3.4 DPM opportunities (DPMO) and is considered “World Class Quality.” The six sigma method allows for universal quantitative comparison of diverse auto analyzers and also enables comparison among various laboratories along with their methodologies. Errors in the laboratory can be minimized if the six standard deviations between the mean of a test along with the upper and lower limits of the test are preserved [8]. In the present study, PAEs in relation to age and gender were found to be 1.11% and 0.27%. The results were in accordance with Kulkarni et al. wherein in their study it was found to be 0.31% and 0.28% respectively [14]. The PAEs in relation to clotted samples were found to be 0.94%. The results were in accordance with Hjelmgren et al., Rooper et al., and Salvagno et al. [15-17]. In the present study, PAEs in relation to incorrect vials were found to be 0.44%. The results were in accordance with Oguz et al. showed a very low PAE frequency of 0.78% in 565,409 samples over a one-year period [18]. Clotted samples and improper collection in vials were the most common PAEs accounting for the majority of PAEs. Clotted/inadequate samples are significantly associated with the pre-analytical procedure and are most likely avoidable with basic preparations [19]. It is critical to properly mix blood samples in order to avoid clots. In contrast to other analytical stages, the pre-analytical phase consists primarily of manual procedures and staff competency with varying levels of education and skills [20]. Increased incidence of PAEs in clotted and inadequate samples can be ascribed to the hospital's recurrent usage of capillary sampling and it seems to be a simple and quick procedure. However, it would be interesting to see if increasing the use of venous samples could minimize the frequency of PAEs [21]. Plebani et al. published QIs with respect to Sigma ratings on a global scale. QIs are universally acceptable tools to estimate the quality of laboratory services. QIs are scientific, objective, measurable, and reproducible tools that are essential for quality management. In comparison to their findings, in the present research QIs had decreased sigma ratings for clotted and for improperly filled samples indicating a larger rejection of blood samples [22]. Numerous studies have reported practical approaches for lowering PAEs, for example, implementation of a collecting module and training classes for the staff. These interventions had an effect in lowering PAEs in the departments which were assessed [16,18,19]. Dorotic et al. noted awareness of PAEs among nurses with regard to hemolysis and noted that the majority of the nurses were unaware of the root cause of PAEs and the subsequent implication of PAEs on laboratory tests [20]. Personnel who are not following pre-analytical protocols that can be attributed to inexperience and incompetence or because of working in stressful environment. Moreover, Increased work turnover and lack of formal education can compromise the blood sample collection [21]. Blood sampling on a regular basis is difficult and can be traumatizing especially in cases of children and their families. So, evaluation of PAE process necessitates careful handling of samples and test requisition forms in order to provide adequate patient care and further obtain high-quality blood samples [22,23]. And also for healthcare practitioners, duplicated sampling is expensive and protracted [24]. Most of the laboratory errors occur in the pre-analytical phases due to person/system design deficiencies. Such problems can be reduced with adequate training and proper sensitization of the paramedical staff particularly during collection and transportation of specimens. In order to minimize the risk of errors sample received in lab should be carefully screened for completeness of requisition form with regards to name, age, sex, Unique hospital identification number, inpatient/outpatient, clinicians name with signature, clinical diagnosis, date and time of sample collection and also quality of the samples (hemolysed/clotted/lipemic/insufficient samples/inappropriate vials) must be assessed.

Limitations

The collation of data on the number of blood specimens from arterial, capillary, and venous blood sampling was lacking in a laboratory information system. There were also no particulars on how many PAEs were extracted from blood samples drawn from central venous lines, which might have caused misleading increased and reduced values. The vast number of observations collected throughout the course of the eight-year study was one of the study's strengths. To our knowledge, this is the first kind of research conducted in South India that mainly focused on PAE especially in hematology section.

## Conclusions

The current study noted that the prevalence of PAEs especially during the collation of blood sample was increased which frequently impact the hematology laboratory owing to clots and insufficient samples. The overall pre-analytical process had a six-sigma score of 3.9, which is barely adequate and highlights the need for improvement. Current research findings should urge medical professionals and laboratory personnel to collaborate in order to lower the frequency of PAEs by concentrating on the upgraded blood collection practices and protocols. As pre-analytical factors constitute the bulk of the laboratory errors our institute has initiated a special phlebotomy certificate course where paramedical staff in association with the Skill lab are exclusively provided in-house training, on the correct procedure to be followed regarding sample collection transport storage, and dispersal of reports as per the SOP which, in turn, have been framed in compliance with National Accreditation Bodies. The present research focuses on the safety of patients and emphasizes decreasing repeated withdrawal of blood which gives agonizing experience to the patients and their families. Continuous monitoring of quality parameters along with route cause analysis of any discrepancies detected along with initiation of remedial measures will go a long way in the prevention of laboratory errors and improving the quality of services.
